# Determination of Lead in Fruit Grown in the Vicinity of Tailings Dams of a Mine in Zacatecas, Mexico

**DOI:** 10.3390/toxics13030188

**Published:** 2025-03-06

**Authors:** Verónica Ávila Vázquez, Miguel Mauricio Aguilera Flores, Agali Naivy Veyna Robles, Lilia Elizabeth Solís Lerma, Omar Sánchez Mata, Sergio Miguel Durón Torres

**Affiliations:** 1 Interdisciplinary Professional Unit of Engineering, Campus Zacatecas, Instituto Politécnico Nacional, Blvd. del Bote 202 Cerro del Gato Ejido La Escondida, Col. Ciudad Administrativa, Zacatecas 98160, Mexico; vavila@ipn.mx (V.Á.V.); omsanchezm@ipn.mx (O.S.M.); 2 Academic Unit of Chemical Sciences, Autonomous University of Zacatecas, Carr. Zacatecas-Guadalajara Km. 6, Ejido la Escondida, Zacatecas 98160, Mexico

**Keywords:** anodic stripping voltammetry, Codex Alimentarius, foliar absorption, fruit contamination, health risks, lead concentrations, Mexican standards, radicular absorption

## Abstract

This study analyzed the lead concentrations in fruit grown near tailings dams of a mine in Zacatecas (Mexico) using electrochemical techniques. A 3 × 4 factorial design, with three levels of apple tree distance (low, medium, and high) and four levels of apple tree part (stem, leaf, pulp, and peel), was performed to predict the pathway for contamination (foliar or radicular). Samples of each apple tree part, soil, and irrigation water were collected. The lead concentrations were determined by anodic stripping voltammetry. The results showed lead concentrations of 172 ppm and 0.012 ppm for the soil and irrigation water, which were discarded as sources of contamination since they were below the allowable limits by the Mexican standards (400 ppm and 2 ppm, respectively). However, lead concentrations in the stem and leaf ranged from 6.6 ppm to 30.7 ppm, and pulp and peel exceeded 300 times the allowable limit by the *Codex Alimentarius* (0.1 ppm). The apple tree part was a significant factor in the experimental design. Hence, it was predicted that the pathway for contamination is by foliar absorption. The fruit is highly contaminated by its proximity to the mine. Therefore, mitigation actions must be performed to avoid health risks for the consumers of this fruit.

## 1. Introduction

Mining is one of the main economic sectors in Mexico that generates social benefits such as employment, foreign exchange, and cultural development. Mexico has remained one of the leading mining countries globally, ranking among the ten highest producers of 16 metals and minerals in 2022. This sector contributed 2.46% to the national Gross Domestic Product [1]. However, this activity has considerable negative impacts on the environment since it generates elevated amounts of waste (tailings dams), causing loss of biodiversity and vegetation cover, mass destruction of water bodies, land-use changes, air contamination, social conflicts, a high cost of living, and food insecurity [2,3].

Tailings dams are the principal source of contamination by heavy metals since they contaminate the soil, water, and air, and the heavy metals contained in them could be bioaccumulated and biomagnified in the food chain [2,4,5]. Arsenic, cadmium, chromium, mercury, and lead rank among the priority metals due to their high toxicity degree and public health significance [4,6]. The United States Agency for Toxic Substances and Disease Registry classified these elements as human carcinogens and mutagens even at low levels of exposure [7]. Lead is extensively used in mining and other industrial processes. It is not easy to give up its use due to its properties. However, this element is non-biodegradable, and its continuous use accumulates its concentration in the environment with increasing hazards [8]. Therefore, lead has been considered a threat to human health [9,10].

Lead is released into the air by mines and factories, transferred to soil and surface water by rainwater, and introduced into vegetable and fruit cultivation by contaminated water, air, and soil [10,11]. Hence, humans, animals, and plants bioaccumulate this element when they coexist in a contaminated environment [2]. Lead is easily absorbed in the human body as a calcium analog, and children are more vulnerable than adults [10]. The World Health Organization recommends a blood lead concentration of 5 μg/dL as a trigger for a thorough review of how a person is being exposed to lead and for action to reduce or end this exposure based on the *Guideline for Clinical Management of Exposure to Lead* [12]. Lead exposure causes inflammation and cardiovascular, digestive, respiratory, neurologic, and urinary diseases [8].

On the other hand, the absorption of lead by plants is generally the first step in incorporating it into the food chain. Plant species obtain lead from irrigation water, the atmosphere, and soil–root transfer [13,14,15]. Although lead has no biological purpose in plants, few species have been reported as tolerant and hyperaccumulators since they accumulate concentrations of this metal at >1000 µg/g without harmful effects [16]. However, many plants are intolerant to it, showing fast inhibition of growth, blackening of the root system, chlorosis, photosynthesis inhibition, physiological activity disorders, and cell death at high concentrations [17].

It has been observed that several species of plants absorb lead and accumulate it in the roots, and only a minimum fraction is transported to aerial plant parts [18,19]. Therefore, root and tuber crops such as carrots, potatoes, cassava, and curcumin could remove lead from the soil and bioaccumulate it [18,20]. In this sense, agricultural production systems constitute a significant non-point source of heavy metal-type pollutants, and their use facilitates their accumulation in the soil and their transfer into the soil–plant–consumer chain [21]. Several cases of edible products that absorb lead have been found, such as cassava (19.92 mg/kg), curcumin (3.25 mg/kg) [20], beetroots (0.173 mg/kg), tomatoes (0.294 mg/kg), carrots (0.206 mg/kg), celery (0.259 mg/kg) [22], and pumpkin leaves (6.40 µg/g) [23].

Hence, it is necessary to analyze the food products cultivated at sites susceptible to lead contamination. Similarly, it is essential to identify the pathway by which this element enters the plant and fruit and to propose mitigation and control measures based on health and environmental regulations. The Food and Agriculture Organization of the United Nations (FAO) and the World Health Organization (WHO) issued the *Codex Alimentarius* (“Food Code”), which is a set of standards, guidelines, and codes of practice to protect the health of consumers. This standard establishes the maximum level of heavy metals in food without causing any adverse effects on human health; 0.1 ppm is the maximum lead level in apples [24]. Although Mexico has not emitted a regulation on lead concentration in fruits or crops, the Mexican standards establish a lead concentration limit in irrigation water of 2 ppm [25] and agricultural/residential/commercial and industrial soil types of 400 and 800 ppm, respectively [26].

Various analytical techniques are employed to determine the lead concentrations in different arrays, such as atomic absorption spectroscopy, flow injection analysis, and inductively coupled plasma mass spectrometry. However, these techniques become unattractive since they use expensive instruments that must be specially maintained and require tedious sample preparations, lengthy operational procedures, and unsuitability for online monitoring. Instead, electrochemical techniques, where the analyte is derived from the measurement of the current intensity as a function of the applied potential, show limits of detection in the order of ppb and are low-cost [27].

Therefore, this study aimed to analyze the lead concentrations in fruit grown near tailings dams of a mine in Zacatecas (Mexico) using electrochemical techniques. Furthermore, a 3 × 4 factorial design, with three levels of apple tree distance (low, medium, and high), four levels of apple tree part (stem, leaf, pulp, and peel), and two replicates, was performed to predict the pathway for contamination (foliar or radicular absorption). The results obtained could aid in taking actions based on the “Food Code” for the prevention and reduction of health risks of lead in consumers of this product.

## 2. Materials and Methods

An orchard where apple trees grow near tailings dams of a mine in Zacatecas (Mexico) was selected as a case study to determine lead concentrations. This mine operated from the 1950s until February 2006. It was subsequently closed for more than 11 years due to a strike. Then, it was rehabilitated in August 2018, and copper, silver, zinc, and lead were extracted. This study was conducted in three stages. The first stage implicated sample collection and the treatment of irrigation water, soil, stems, and leaves of apple trees, and apple pulp and peel. Sampling was carried out in September 2019. The apple harvest takes place mainly between July and October in Mexico, and September is usually the month with the highest production [28]. The second stage involved determining the lead concentrations in all samples. This stage was performed by electrochemical technique, specifically anodic stripping voltammetry (ASV), to determine the lead concentration in the different matrices. The third stage considered a statistical analysis of the results to predict if the pathway for contamination is by foliar or radicular absorption.

### 2.1. Stage 1. Sample Collection and Treatment

**Figure 1:**
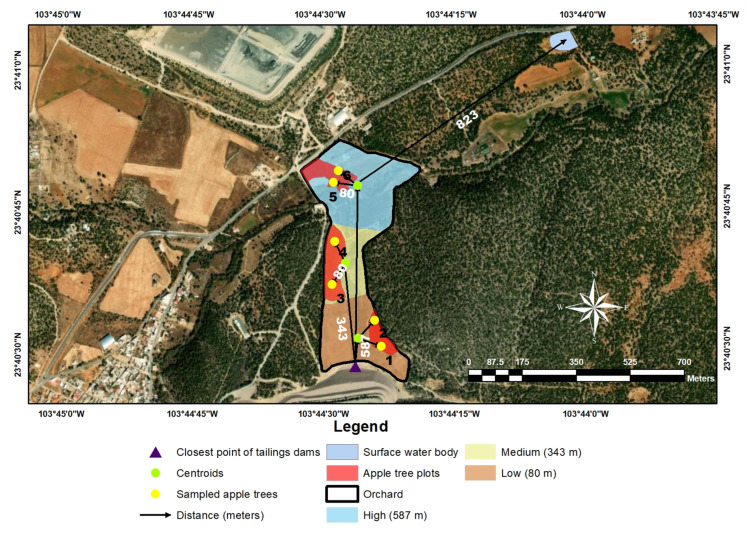
Map of the study area.

A representative water sample was taken from a surface water body used to irrigate apple trees. The surface water body is intermittent and is 0.8 km from the nearest apple orchard (Figure 1). The sample was obtained using a lead-free container immersed for two hours in nitric acid (at 3% *v*/*v*) and thoroughly rinsed with deionized water. The container was rinsed three times with irrigation water. Then, the sample was collected and stored at 4 °C until its use. The water sample was filtered to remove suspended particles and treated according to *Method 3051A Microwave Assisted Acid Digestion* with the following modification [29]. A total of 5 mL of nitric acid (at 65% *v*/*v*) and 5 mL of deionized water were added for sample treatment. This adjustment was performed to avoid electrode pitting due to the medium acidity [27].

A 3 × 4 factorial design with three levels of apple tree distance (low, medium, and high), four levels of apple tree part (stem, leaf, pulp, and peel), and two replicates were selected to study the effect of the distance between the apple trees and tailing dams of the mine (factor 1) and the tree part where it accumulates the lead (factor 2), as shown in Table 1. The lead concentration was the response analyzed in the experimental design.

Figure 1 shows the map of the study area where the sampling was carried out on the apple trees surrounding the tailings dams of the mine. Three apple crop plots were identified, taking the centroids of the area of each crop plot as a reference point (low, medium, and high) based on their distance from the tailings dams. Two apple trees 80 m away from the centroid in each crop were selected to take the samples. Therefore, samples of the stem, leaf, pulp, and peel of the apple trees chosen were collected according to the experimental design. Likewise, soil samples where apple trees grow were collected to rule out whether the contamination is foliar or reticular absorption. The distances for taking the samples were ensured to be as precise as possible.

Stems, leaves, and fruit were collected from each studied apple tree. The peel and pulp were separated from the fruit. The samples were cleaned with tap water and phosphate-free soap. Then, they were washed first with distilled water and deionized water. Later, the samples were dried at 70 °C in a convective flow stove FELISA FE-291AD (Feligneo, Zapopan, Mexico) for 48 h. Afterward, they were macerated with a mortar and placed in polyethylene bags at room temperature until digestion. The digestion was performed according to *Method 3051A Microwave Assisted Acid Digestion* with the mentioned modification [27,29].

The soil samples collected were associated with the apple tree selected in the collection of the samples. A soil sample of approximately 1 kg was taken from the closest part of the trunk of each sampled tree. A soil sampling auger was used, which allowed the soil to be probed to a depth of at least 15 cm. The soil samples were stored in polyethylene bags based on the Mexican Standard NMX-AA-132-SCFI-2016 [30]. Then, they were placed in aluminum trays and dried at 60 °C in a convective flow stove FELISA FE-291AD (Feligneo, Zapopan, Mexico) to remove the moisture. Then, they were sieved at 500 microns and placed in polyethylene bags at room temperature until their digestion. The digestion was performed according to *Method 3051A Microwave Assisted Acid Digestion* with the mentioned modification [27,29].

An apple tree and soil samples from a residential house were taken as blanks in Chalchihuites, Zacatecas, Mexico, remote from the study area (at 50 km) and without perceptible lead contamination sources. The site’s geographic coordinates were—103.898931 West longitude and 23.396635 North latitude. The samples were equally treated and analyzed as described. This site was selected for the blank samples because it does not have an apparent source of lead contamination, and it would serve to validate the electrochemical techniques and compare the results between the blank samples and study matrices.

### 2.2. Stage 2. Measuring the Lead Concentrations in Matrices by Electrochemical Techniques

**Figure 2:**
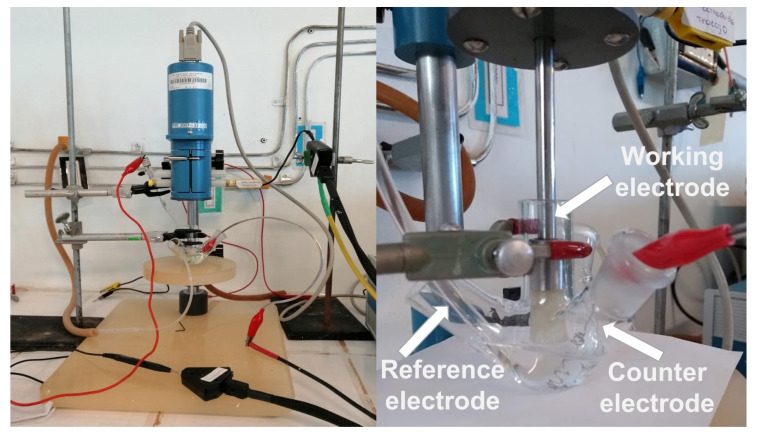
Three-electrode system for anodic stripping voltammetry analysis.

Lead concentrations in the stem, leaf, peel, pulp, and irrigation water were measured by the electrochemical technique of differential pulse anodic stripping voltammetry (DPASV) and the soil samples by linear potential sweep anodic stripping voltammetry (LSASV), using a Potentiostat/Galvanostat Model 283 (AMETEK, Oak Ridge, TN, USA) and a Rotating Ring-Disk Electrode System Model 636 (AMETEK, Oak Ridge, TN, USA). Glassy (vitreous) carbon, saturated calomel, and platinum were used as the working, reference, and counter electrodes (Figure 2).

The limit of detection (LoD) and limit of quantitation (LoQ) were estimated using the standard addition method for each technique applied. This method consisted of adding aliquots of 1 or 0.5 microliters (μL) (depending on whether the initial current intensity was high or low) of a stock solution of lead (1000 ppm) in 2 wt% nitric acid (Sigma-Aldrich, San Luis, CA, USA). A standard curve for the low concentration was prepared from the lead stock standard (J.T. Baker, Phillipsburg, NJ, USA) to 0.1, 0.25, 0.5, 0.8, and 1 ppm. The estimated limits are shown in Table 2.

All the experiments were performed using a three-electrode conventional electrochemical cell fabricated with glass and a capacity of 10 mL (Figure 2). The solutions were prepared with a ratio of 1:2 (sample: cell volume), using 5 mL of amalgamating solution [100 ppm of mercury (II) nitrate monohydrate (J.T. Baker, Phillipsburg, NJ, USA) and 0.2 M potassium nitrate solution (Sigma-Aldrich, San Luis, CA, USA), gauged with nitric acid 10% *v*/*v* (J.T. Baker, Phillipsburg, NJ, USA)], and 5 mL of the digested sample. Each sample was placed in a process of purging with nitrogen gas for 10 min.

The lead concentration measurements by DPASV were performed using the standard addition method. This method consisted of adding aliquots of 1 or 0.5 μL (depending on whether the initial current intensity was high or low) of a stock solution of lead (1000 ppm) in 2 wt% nitric acid (Sigma-Aldrich, San Luis, CA, USA) until obtaining 3 to 5 additional peaks concerning the initial concentration peak. The operating conditions consisted of forming a mercury film and depositing the problem ions on the working electrode surface, applying a potential of −1.2 V for 435 s and agitation at 900 rpm. Then, the agitation was stopped for 45 s to homogenize the mercury amalgam. Subsequently, a sweep from −0.6 V to −0.4 V was applied at a velocity of 20 mV/s (a pulse amplitude of 5 mV every 250 ms), with pulse height and width of 50 mV and 50 ms, respectively. Each sample was analyzed in duplicate.

The lead concentration measurements by LSASV were performed similarly. For this case, 5 mL of amalgamating solution, 1.25 mL of digested sample, and 3.75 mL of deionized water were added. A sweep from −1.2 to 0.3 V was applied at a velocity of 20 mV/s (a pulse amplitude of 5 mV every 250 ms). Then, the agitation was stopped for 45 s. Each sample was analyzed in duplicate.

**Figure 3:**
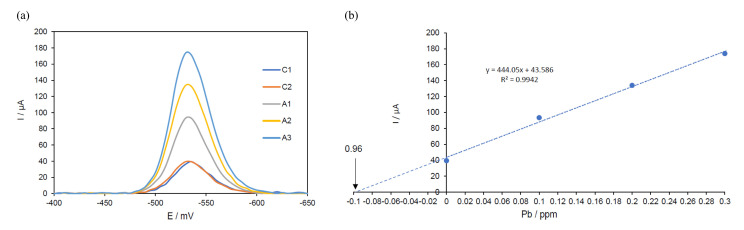
(**a**) Voltammograms of the leaf sample (assay number 6), where C1 and C2 are the initial sample readings and A1, A2, and A3 are the lead additions. (**b**) The maximum current intensity versus the lead concentration in the leaf sample (assay number 6).

The results obtained in the voltammograms were analyzed with a data analysis program using the Origin Pro 8.0 software (OriginLab, Northampton, MA, USA). The baseline of the curves was eliminated. The maximum current intensity of the samples and the additions were plotted concerning the concentrations of the added aliquots of the stock solution of lead, resulting in a graph with an exponential growth trend (Figure 3a). Then, a linear regression of the data of the maximum current intensity obtained in the voltammogram versus the lead concentrations of each added aliquot was performed (Figure 3b). The value that is intersected with the ordinate corresponds to the sample concentration without dilution factors. Finally, the obtained value was multiplied by the dilution factors (125 of the digestion and 2 of the cell) to obtain the lead concentration.

### 2.3. Stage 3. Statistical Analysis of the Results

An analysis of variance (ANOVA) was performed from the experimental design using Design-Expert^®^ version 12 software (Trial version) (Stat-Ease, Inc., Minneapolis, MN, USA). The significance of the model, the factors, and the lack of fit were established at a *p*-value of <0.05 [31]. The prediction of the pathway for contamination (foliar or radicular absorption) was determined from the results of the lead concentration, significant factors in the ANOVA, and environmental conditions (the elevation profile and predominant wind direction).

## 3. Results and Discussion

### 3.1. Lead Concentrations in the Different Matrices

Table 3 shows the lead concentrations in the stem, leaf, pulp, and peel of the apple trees and soil.

On the one hand, lead concentrations between 6.6 ppm and 18.3 ppm were found in the stems of the apple trees, with the averages based on the distance of 15.5 ppm (low), 14.5 ppm (medium), and 9.2 ppm (high) (Table 3). Lead concentrations between 9 ppm and 15.5 ppm were found in the pulps of the apple, with the averages based on the distance of 15 ppm (low), 13.7 ppm (medium), and 9.7 ppm (high) (Table 3). The distance between the apple trees and the tailings dams of the mine could be a factor influencing the lead concentration in the apple trees since the higher lead concentrations were identified in apple trees of the low zone (80 m of tailings dams) and the lower ones in the high zone (587 m of tailings dams).

On the other hand, the highest lead concentrations were found in the leaves of the apple trees and the peels of the apples (Table 3). Lead concentrations between 16.7 ppm and 30.7 ppm, with averages based on the distance of 19.1 ppm (low), 26.1 ppm (medium), and 21.8 ppm (high), were found in the leaves of the apple trees (Table 3). Lead concentrations between 11.3 ppm and 30.4 ppm were found in the peels of the apples, with averages based on the distance of 15.5 ppm (low), 19.8 ppm (medium), and 28 ppm (high) (Table 3). It is noted that the distance between apple trees and the tailings dams of the mine was not a factor influencing the lead concentration since the higher lead concentration was estimated in the apple trees of the medium zone (at 343 m of tailings dams). Therefore, the apple tree distance (low, medium, and high) and apple tree part (stem, leaf, pulp, and peel) were the factors studied in the experimental design, evaluating their influence on the response (lead concentration) in the analysis of variance ANOVA.

Lead concentrations between 135 ppm and 172 ppm were found in the soil samples, with the averages based on the distance of 148 ppm (low), 168 ppm (medium), and 141 ppm (high) (Table 3). It can be noted that there is no clear trend in which the lead concentration has a direct relationship with the distance between apple trees and the tailings dams of the mine. The Mexican Standard NOM-147-SEMARNAT/SSA1-2004 establishes criteria for determining the remediation concentrations of the soils contaminated by lead and other metals, specifying lead reference values of 400 ppm and 800 ppm for agricultural/residential/commercial and industrial use [26]. None of the soil samples exceeded the reference value for soil of agricultural use. Therefore, the soil of the apple tree plots does not require remediation for lead contamination. However, the soil of the apple tree plots has been contaminated since lead is naturally present in all soils at concentrations of 15 to 40 ppm [32], exceeding these values up to 4.3 times the analyzed soil samples in this study. Hence, tailings dams of the mine could be a contamination source of lead in these apple tree plots since lead is one of the minerals extracted by the mine.

Additionally, future contamination should not be ruled out if the control techniques are not maintained at the tailings dams since Félix et al. [27] characterized the soil around the tailings dams of the mine in this study in 2018, finding lead concentrations of 1115 ppm. This value was 2.87 times higher than the reference value stipulated in the Mexican Standard [26]. Therefore, these results suggest that the lead is dispersed by wind erosion and chemical wear of the tailings dams until it reaches the apple tree plots.

Irrigation water was taken from a surface water body and was discarded as a contamination source of lead in the soil of the apple tree plots since a lead concentration of 0.012 ppm was found. This value was 416.7 and 166.7 times lower than the maximum concentrations for irrigation stipulated by the United States Environmental Protection Agency (5 ppm) [33] and Mexican Standard NOM-001-SEMARNAT-2021 (2 ppm) [25]. Apple trees are irrigated weekly with this water. However, this practice is considered suitable since it does not represent a contamination source due to the very low lead concentration reported in this study.

The samples, considered blanks, showed 0.012 ppm, 0.100 ppm, 0.013 ppm, and 0.090 ppm in the stem, leaf, pulp, and peel, respectively. The average values of the study area exceeded 13.1, 22.3, 12.8, and 21.1 times the blank values, respectively. Likewise, the blank soil sample showed a lead concentration of 24 ppm. The average value of the soil samples of the apple tree plots was 152.3 times higher than the blank value. Therefore, it is evident that the apple tree plots are strongly contaminated by lead. The results suggest that the leaves and peels of apple trees have a higher capacity to accumulate this contaminant.

The Food and Agriculture Organization of the United Nations and the World Health Organization emitted the General Standard for Contaminants and Toxins in Food and Feed through the *Codex Alimentarius CXS 193-1995*, stipulating a maximum lead level in apples of 0.1 ppm [24]. The apples analyzed in this study exceeded 128 and 211 times this limit, considering average values, and the highest values in the apple peel and pulp samples exceeded 245 and 300 times, respectively. Hence, fruit represents health risks related to lead in consumers of this product.

Table 4 shows studies on lead accumulation in crops near contaminated soils.

García-Gallegos et al. [34] found that oat and broad bean crops can absorb lead from the soil through their roots and translocate it to the aerial part (stems and leaves), showing tolerance to the contamination by lead. They demonstrated that the plant height, root volume, and total dry biomass are not affected by lead contamination. Therefore, the plants did not show any phytotoxic effects by direct contact with lead-contaminated soil. However, Ruiz-Huerta et al. [35] also studied the accumulation of arsenic and heavy metals (among them lead) in maize near mine tailings. They identified visible affectations in the plant, such as chlorosis, thinner leaves, and growth inhibition. In addition, lead was one of the heavy metals with the highest concentration in maize (Table 4). Therefore, comparing both studies with the results obtained in this work, it is evident that plants cultivated in zones close to tailings dams of the mines accumulate heavy metals, causing visible and non-visible toxic effects.

Zhuang et al. [36] investigated the contamination levels of lead in soils, vegetables, and rice grown near the Dabaoshan mine in China. Generally, the vegetables did not exceed the maximum permissible level recommended for fresh-leaf vegetables in China (Table 4). However, the lead concentrations in different parts of the rice plant were in the order of straw > hull > grain, with average values of 16 ppm, 5 ppm, and 2 ppm, respectively. The values exceeded 7.2 times the permissible values (0.2 ppm) for cereals in China. The authors [36] did not predict the pathway for contamination. However, they highlighted the importance of investigating the status of heavy metal concentrations in food crops grown near tailings dams.

Liu et al. [37] studied the heavy metal contamination of soils and crops affected by a Chenzhou lead/zinc mine in Hunan, China. Cereal (rice, maize, and sorghum), pulses (soybean, Adzuki bean, mung bean, and peanut), vegetables (ipomoea, capsicum, taro, and string bean), and the rooted soils were the matrices studied. In general, edible leaves or stems of crops showed higher contamination than seeds or fruits. Ipomoea was the most severely contaminated crop with lead (Table 4), finding concentrations in its leaves 8.5 times higher than the maximum permitted level (9 ppm). The results coincide with those obtained in this work, where apple tree leaves showed the highest concentrations (average values of 22.3 ppm, 21.1 ppm, 13.1 ppm, and 12.8 ppm in leaves, peels, stems, and pulps, respectively).

Bioconcentration and translocation factors have been proposed to demonstrate the behavior of heavy metals in plants. The bioconcentration factor evaluates the content of heavy metals in the plant. It is calculated by dividing the metal concentration in the plant part by the metal concentration in the soil. The translocation factor measures the quantity of the heavy metals transferred from one organ to another. It is calculated by dividing the metal concentration in the plant shoot by the metal concentration in the plant root and shoot systems [39]. Bioconcentration factors were calculated for this study, showing values of 0.45 and 0.43, 0.47 and 0.41, and 0.49 and 0.47 for the low, medium, and high zones, respectively. These values are less than 1, indicating more lead in the environment than in the apple trees [39]. Therefore, the lead in the environment is again associated with mine waste (tailings dams) as a contamination source.

The translocation factors could not be calculated since the lead concentration in apple tree roots was outside the scope of this study. However, it could be analyzed as future work to rule out that apple trees are hyperaccumulating plants and act as phytoremediators of the contaminated site. It is possible that the translocation factors would also result in values less than one based on the values obtained in the bioconcentration factors. Therefore, apple trees would be ruled out as hyperaccumulators or phytoremediators.

### 3.2. Statistical Validation

Table 5 shows the analysis of variance ANOVA of the 3 × 4 factorial design.

It can be noted that the model of the experimental design is significant according to the analysis of variance ANOVA since the *p*-value is less than 0.05 (Table 5). The distance between apple trees and the tailings dams of the mine (factor 1) turned out to be a non-significant factor since its *p*-value is higher than 0.05 (Table 5). This result coincides with the results shown in Table 3, where it was demonstrated that the distance between apple trees and the tailings dams of the mine did not influence the lead concentration since the higher lead concentration was estimated in apple trees of the medium zone (at 343 m of tailings dams).

On the other hand, the apple tree part (factor 2) is a significant factor since its *p*-value is less than 0.05 (Table 5). The different parts of the apple trees showed average values of lead concentration of 22.3 ppm, 21.1 ppm, 13.1 ppm, and 12.8 ppm in the leaves, peels, stems, and pulps, respectively, from highest to lowest, highlighting significant differences in the average value of each part of the apple trees. Likewise, the interaction of both factors (factor 1 × factor 2) is also significant, with a *p*-value less than 0.05. Therefore, combining these two factors influences the lead concentration in apple trees. Although the main effect of distance was not significant, it becomes relevant when interacting with the tree part. Hence, the distance between apple trees and the tailings dams of the mine could not be completely ruled out. Therefore, the pathway for contamination (foliar or radicular absorption) was analyzed and predicted.

### 3.3. Pathway for Contamination (Atmospheric Deposition)

**Figure 4:**
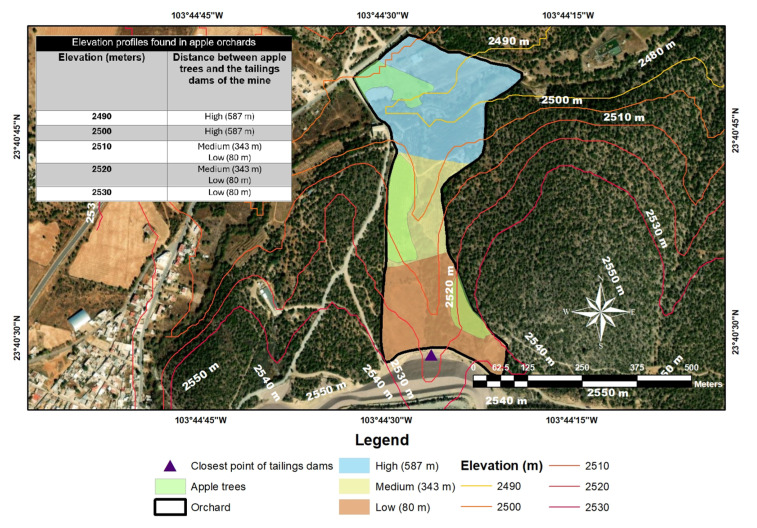
Elevation profile of the study area.

Figure 4 shows the elevation profile from the tailings dams of the mine to the apple orchard. It can be observed that the average elevation profiles of the tailings dams and apple orchards are 2530–2550 and 2490–2530 m above sea level, respectively. Although the elevation difference between the tailings dams and the apple orchard would promote the conditions for leaching heavy metals in the soil, the orography as a climatic factor of the terrain prevents leachates from moving towards the apple orchard, generating a natural barrier that surrounds the surface of the nearby soil between the crop area and the mine tailings.

Wang et al. [38] analyzed the contamination of farmland and orchards with heavy metals close to the tailings dams of an abandoned lead–zinc mine in Zixing City, China. The residents primarily focus on cultivating fruit trees for commercial purposes, with cultivating vegetables and staple crops as a secondary activity. However, the design and construction of the tailings dams lacked proper standards. Wang et al. [38] predicted that contamination is mainly caused by the migration and diffusion of the metals downstream since tailings dams were in the upstream region. This condition did not apply to the study area of this work.

**Figure 5:**
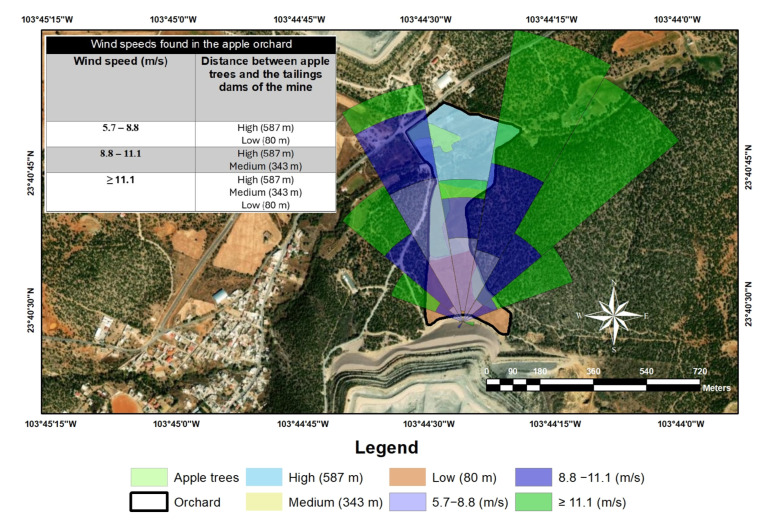
Predominant wind direction of the study area.

Figure 5 shows the predominant wind direction of the study area. It can be noted that the flow vector of the predominant wind direction that goes from the tailings dams to the apple orchard is North-Northeast (NNE) and North (N), with an average velocity between 5.7 and ≥11.1 m/s. Both climatic elements (direction and velocity) promote the conditions for a possible wind dispersion of particles to the crop plot. Based on the results of the ANOVA, shown in Table 5, the distance between apple trees and the tailings dams was not a significant factor. Hence, fruit contamination could be attributed to the dispersion of particulate material with lead content towards the apple orchard plots (atmospheric deposition) caused by the predominant wind direction and velocity (Figure 5). Therefore, the pathway for contamination was predicted as foliar absorption.

Zhu et al. [40] compared the mechanisms of heavy metal absorption from soil in the leaves and roots of different crops. They identified that the highest absorption is in the roots. However, they also found that there is foliar absorption of heavy metals present in the air. The highest heavy metal concentrations in the leaves were lead, cadmium, arsenic, and chromium. According to this study, it can be associated that the pathway for lead contamination of the apple trees (leaves and fruit) is by atmospheric deposition (wind erosion and chemical wear of the tailings dams of the mine). Therefore, it is verified that lead accumulation in apple trees is caused by foliar absorption.

An evaluation of the influence of the seasonal period on lead concentrations found in apple trees was outside the scope of this study. However, it is essential to consider it as future work. This study was carried out during the months of rainfall on the site (September 2019 presented a precipitation of 69 mm [41]), so this factor could have favored the atmospheric deposition of lead on the aerial part of apple trees and promoted the absorption and accumulation of this heavy metal. Yu et al. [42] demonstrated that atmospheric deposition by sedimentation or dragging of raindrops positively impacts heavy metal enrichment in crops. Therefore, the precipitation was a factor that affected the lead concentrations found in the apple trees of this study.

### 3.4. Mitigation Measures in the Control of Contamination by Lead

Use of natural barriers as endemic trees to break or interrupt winds, as well as chemical or biological stabilization with biosolids, the use of synthetic or vegetative covers (phytostabilization), protection of the surface with recovered soil or materials that allow the fixation of plant species native to the region, and carrying out reforestation and restoration programs to stabilize the slopes of the main barrier to keep the surfaces protected from erosion by wind and rain action are the mitigation measures proposed in the control of tailings dams of the mine. These measures could prevent solid particles from being emitted into the atmosphere caused by the loss of moisture from the tailings dams’ surface or the slope of the containment curtain and the formation of runoffs that affect surface and underground water bodies [43].

The Food and Agriculture Organization of the United Nations (FAO) and the World Health Organization (WHO) established the Code of Practice CAC/RCP 56-2004 for the prevention and reduction of the presence of lead in foods and to reduce its content in crops [44]. The proposed recommendations are outlined below.

- Periodically monitor the lead content in soils near crop plots and in the crop plots to discharge that which exceeds the limits established in the environmental regulations.
- Do not use compounds containing lead, such as pesticides based on lead arsenate, or substances that could be contaminated with lead, such as copper fungicides or improperly prepared phosphate fertilizers.
- Do not use machines or equipment that use gasoline with lead as fuel, such as dryers.
- The product (apples) must be protected from lead contamination, for example, exposure to lead from air, soil, or water contamination.
- Organic and inorganic amendments such as compost, biosolids, or manure can be incorporated into the soil, and protective sheets can be used to reduce soil contact deposition on trees and to prevent lead from becoming available to trees.
- Water for irrigation could be protected from sources of lead contamination, and its lead content must be monitored to prevent or mitigate contamination of crops by lead.
- Periodically analyze the lead content in the fruit (apples) to ensure that its concentration does not exceed the limits established by the environmental regulations.

It is essential to ensure the safety and stability of tailings dams, controlling the environmental impacts under the applicable regulations since their omission can cause severe damage to the population and the environment and high economic losses. Such was the case of the collapse of the tailings in Guangdong, China, in 2010. This event caused economic losses of 460 million yuan, the death of 22 people, damage to 6370 houses, and an affected crop area of 72.6 km^2^. The causes were associated with the climatological conditions (heavy rain) and neglectful work of the mining departments (design, supervision, and construction) [45]. Therefore, government authorities must pay attention to this activity to avoid damage to the environment that causes high economic losses and damage to the population by lack of control and stability of the tailings dams.

## 4. Conclusions

The results showed the possibility of potential lead exposure in consumers of apples grown in the vicinity of the tailings dams of a mine in Zacatecas (Mexico) since the apple peel and pulp samples exceeded 245 and 300 times, respectively—the allowed limit by the *Codex Alimentarius* (0.1 ppm). The lead concentrations in soil (from 135 ppm to 172 ppm) and irrigation water (0.012 ppm) were below the allowed limits by the Mexican standards NOM-147-SEMARNAT/SSA1-2004 (<400 ppm) and NOM-001-SEMARNAT-2021 (2 ppm), respectively, being discarded as contamination sources. However, lead concentrations in the stem and leaf from 6.6 ppm to 30.7 ppm were found, predicting that the pathway for contamination is by foliar absorption due to the wind dispersion of tailings dams that arrive at the apple orchard. Therefore, the results of this study provide information on the contamination by lead in fruit grown near tailings dams of a mine and the possibility of human exposure to lead. In addition, the results support taking actions based on the Code of Practice CAC/RCP 56-2004 for preventing and reducing lead contamination in foods emitted by the FAO/WHO.

## Figures and Tables

**Table 1:** The 3 × 4 factorial design used in this study.

Assay Number	Factor 1: Apple Tree Distance ^1^	Factor 2: Apple Tree Part	Apple Tree Number ^2^
1	Low	Stem	1
2	Medium	Stem	3
3	High	Stem	5
4	Low	Leaf	1
5	Medium	Leaf	3
6	High	Leaf	5
7	Low	Pulp	1
8	Medium	Pulp	3
9	High	Pulp	5
10	Low	Peel	1
11	Medium	Peel	3
12	High	Peel	5
13	Low	Stem	2
14	Medium	Stem	4
15	High	Stem	6
16	Low	Leaf	2
17	Medium	Leaf	4
18	High	Leaf	6
19	Low	Pulp	2
20	Medium	Pulp	4
21	High	Pulp	6
22	Low	Peel	2
23	Medium	Peel	4
24	High	Peel	6

^1^ Distance between tailings dams of the mine and the study area centroids: low (80 m), medium (343 m), and high (587 m). ^2^ The sampled apple tree location is shown in Figure 1 with black numbers (1–6).

**Table 2:** Limit of detection (LoD) and limit of quantitation (LoQ) for the electrochemical techniques.

Electrochemical Technique	LoD (ppm)	LoQ (ppm)
DPASV ^1^	0.002	0.002
LSASV ^2^	1.530	3.850

^1^ DPASV: differential pulse anodic stripping voltammetry. ^2^ LSASV: linear potential sweep anodic stripping voltammetry.

**Table 3:** Lead concentrations in apple trees and soil.

Assay Number	Factor 1: Apple Tree Distance ^1^	Factor 2: Apple Tree Part	Lead Concentration (ppm)	
			Apple Tree	Soil
1	Low	Stem	15.0	135
2	Medium	Stem	18.3	172
3	High	Stem	6.6	142
4	Low	Leaf	16.7	135
5	Medium	Leaf	21.5	172
6	High	Leaf	24.2	142
7	Low	Pulp	15.5	135
8	Medium	Pulp	13.2	172
9	High	Pulp	9.0	142
10	Low	Peel	13.9	135
11	Medium	Peel	28.3	172
12	High	Peel	30.4	142
13	Low	Stem	16.0	161
14	Medium	Stem	10.7	164
15	High	Stem	11.7	140
16	Low	Leaf	21.6	161
17	Medium	Leaf	30.7	164
18	High	Leaf	19.4	140
19	Low	Pulp	14.5	161
20	Medium	Pulp	14.2	164
21	High	Pulp	10.4	140
22	Low	Peel	7.1	161
23	Medium	Peel	11.3	164
24	High	Peel	25.5	140

^1^ Distance between tailings dams of the mine and the study area centroids: low (80 m), medium (343 m), and high (587 m).

**Table 4:** Studies on lead accumulation in crops near contaminated soils.

Crops	Lead Concentration (ppm)	Reference
Oat	45.3	[34]
Broad bean	55.0	[34]
Maize	66.6–6166.0	[35]
Vegetables	0.1–0.3	[36]
Rice	2.0–16.0	[37]
Ipomoea	76.9	[38]
Apple	6.6–30.7	This study

**Table 5:** Analysis of variance ANOVA of the 3 × 4 factorial design.

Source	Sum of Squares	Degree of Freedom	Mean Square	F Value	*p*-Value
Model	837.31	11	76.12	3.15	0.0302
Factor 1 ^1^	49.38	2	24.69	1.02	0.3891
Factor 2 ^2^	406.01	3	135.34	5.60	0.0123
Factor 1 × Factor 2	381.93	6	63.65	2.64	0.0722
Pure Error	289.83	12	24.15		
Cor Total	1127.15	23			

^1^ Factor 1: Distance between apple trees and tailings dams of the mine. ^2^ Factor 2: Apple tree part.

## Data Availability

The datasets generated and/or analyzed during the current study are available from the corresponding author upon reasonable request.

## References

[1] CAMIMEX Sustainability Report 2023. Mining and Sustainability: A Commitment to Mexico..

[2] Kumar A., Kumar A., Cabral-Pinto M.M.S., Chaturvedi A.K., Shabnam A.A., Subrahmanyam G., Mondal R., Gupta D.K., Malyan S.K., Kumar S.S. (2020). Lead Toxicity: Health Hazards, Influence on Food Chain, and Sustainable Remediation Approaches. Int. J. Environ. Res. Public Health.

[3] Worlanyo A.S., Jiangfeng L. (2021). Evaluating the environmental and economic impact of mining for post-mined land restoration and land-use: A review. J. Environ. Manag..

[4] Ekere N.R., Ugbor M.C.J., Ihedioha J.N., Ukwueze N.N., Abugu H.O. (2020). Ecological and potential health risk assessment of heavy metals in soils and food crops grown in abandoned urban open waste dumpsite. J. Environ. Health Sci. Eng..

[5] Techane G., Sahilu G., Alakangas L., Mulat W., Kloos H. (2023). Assessment of heavy metal pollution associated with tailing dam in gold mining area, southern Ethiopia. Geosyst. Eng..

[6] Tchounwou P.B., Yedjou C.G., Patlolla A.K., Sutton D.J. (2012). Heavy metal toxicity and the environment. Exp. Suppl..

[7] Agency for Toxic Substances and Disease Registry Guidance for the Preparation of Toxicological Profiles. https://www.atsdr.cdc.gov/toxprofiles/guidance/profile_development_guidance.pdf.

[8] Wani A.L., Ara A., Usmani J.A. (2015). Lead toxicity: A review. Interdiscip. Toxicol..

[9] Boskabady M., Marefati N., Farkhondeh T., Shakeri F., Farshbaf A., Boskabady M.H. (2018). The effect of environmental lead exposure on human health and the contribution of inflammatory mechanisms, a review. Environ. Int..

[10] Collin M.S., Venkatraman S.K., Vijayakumar N., Kanimozhi V., Arbaaz S.M., Sibiya Stacey R.G., Anusha J., Choudhary R., Lvov V., Tovar G.I. (2022). Bioaccumulation of lead (Pb) and its effects on human: A review. J. Hazard. Mater. Adv..

[11] Liao X., Li Y., Miranda-Avilés R., Puy-Alquiza M.J., Bian J., Hernández-Anguiano J.H., Serafín-Muñoz A.H., Datta S., Zha X., Liu J. (2023). Assessments of Pollution Status and Human Health Risk of Potentially Toxic Elements in Primary Crops and Agricultural Soils in Guanajuato, Mexico. Water Air Soil. Pollut..

[12] World Health Organization WHO Guideline for Clinical Management of Exposure to Lead. https://iris.who.int/bitstream/handle/10665/347360/9789240037045-eng.pdf?sequence=1.

[13] Bi X., Feng X., Yang Y., Li X., Shin G.P.Y., Li F., Qiu G., Li G., Liu T., Fu Z. (2009). Allocation and source attribution of lead and cadmium in maize (*Zea mays* L.) impacted by smelting emissions. Environ. Pollut..

[14] Uzu G., Sobanska S., Sarret G., Muñoz M., Dumat C. (2010). Foliar Lead Uptake by Lettuce Exposed to Atmospheric Fallouts. Environ. Sci. Technol..

[15] Tong G., Wu S., Yuan Y., Li F., Chen L. (2018). Modeling of Trace Metal Migration and Accumulation Processes in a Soil-Wheat System in Lihe Watershed, China. Int. J. Environ. Res. Public Health.

[16] Reeves R.D., Baker A.J.M., Jaffré T., Erskine P.D., Echevarria G., van der Ent A. (2018). A global database for plants that hyperaccumulate metal and metalloid trace elements. New Phytol..

[17] Nas F.S., Ali M. (2018). The effect of lead on plants in terms of growing and biochemical parameters: A review. MOJ Eco. Environ. Sci..

[18] Kumar B., Smita K., Flores L.C. (2017). Plant mediated detoxification of mercury and lead. Arab. J. Chem..

[19] Collin S., Baskar A., Geevarghese D.M., Ali M.N.V.S., Bahubali P., Choudhary R., Lvov V., Ibrahin Tovar G.I., Senatov F., Koppala S. (2022). Bioaccumulation of lead (Pb) and its effects in plants: A review. J. Hazard. Mater. Lett..

[20] Nobuntou W., Parkpian P., Kim Oanh N.T., Noomhorm A., Delaune R.D., Jugsujinda A. (2010). Lead distribution and its potential risk to the environment: Lesson learned from environmental monitoring of abandon mine. J. Environ. Sci. Health Part A.

[21] Kabata-Pendias A. (2010). Trace Elements in Soils and Plants.

[22] Rusin M., Domagalska J., Rogala D., Razzaghi M., Szymala I. (2021). Concentration of cadmium and lead in vegetables and fruits. Sci. Rep..

[23] Nwachukwu J.I., Clarke L.J., Symeonakis E., Brearley F.Q. (2022). Assessment of human exposure to food crops contaminated with lead and cadmium in Owerri, South-eastern Nigeria. J. Trace Elem. Miner..

[24] Food and Agriculture Organization of the United Nations and World Health Organization General Standard for Contaminants and Toxins in Food and Feed CXS 193-1995. https://www.fao.org/fao-who-codexalimentarius/sh-proxy/es/?lnk=1&url=https%253A%252F%252Fworkspace.fao.org%252Fsites%252Fcodex%252FStandards%252FCXS%2B193-1995%252FCXS_193e.pdf.

[25] Ministry of the Environment and Natural Resources Mexican Official Standard NOM-001-SEMARNAT-2021, Which Establishes the Permissible Limits of Pollutants in Wastewater Discharges in Receiving Bodies Owned by the Nation. https://www.dof.gob.mx/nota_detalle.php?codigo=5645374&fecha=11/03/2022#gsc.tab=0.

[26] Ministry of the Environment and Natural Resources and Ministry of Health Mexican Official Standard NOM-147-SEMARNAT/SSA1-2004, Which Establishes Criteria to Determine the Remediation Concentrations of Soils Contaminated by Arsenic, Barium, Beryllium, Cadmium, Hexavalent Chromium, Mercury, Nickel, Silver, Lead, Selenium, Thallium, and/or Vaadium. https://www.profepa.gob.mx/innovaportal/file/1392/1/nom-147-semarnat_ssa1-2004.pdf.

[27] Félix L.T., Durón S.M., Ávila V., Correa H.C., Aguilera M.M. (2018). Determination of Pb in *Brickellia Veronicifolia* for Anodic Stripping Voltammetry. ECS Trans..

[28] Covarrubias-Ramírez J.M., Parga-Torres V.M., Martínez-Rodríguez J.G., Kahramanoğlu I., Wan C. (2022). Postharvest Management and Marketing of Apples in Mexico. Fuit Industry.

[29] United States Environmental Protection Agency Method 3051A: Microwave Assisted Acid Digestion of Sediments, Sludges, Soils, and Oils. https://www.epa.gov/sites/default/files/2015-12/documents/3051a.pdf.

[30] Ministry of Economy Mexican Standard NMX-AA-132-SCFI-2016, Soil Sampling for Metals and Metalloids Identification and Quantification, and Sample Handling. http://www.economia-nmx.gob.mx/normas/nmx/2010/nmx-aa-132-scfi-2016.pdf.

[31] Montgomery D.C. (2019). Design and Analysis of Experiments.

[32] Center for Agriculture, Food, and the Environment of the University of Massachusetts Amherst Soil Lead Fact Sheet, Soil Lead Contamination. https://ag.umass.edu/soil-plant-nutrient-testing-laboratory/fact-sheets/soil-lead-fact-sheet.

[33] United States Environmental Protection Agency Guidelines for Water Reuse 2012. https://www.epa.gov/sites/default/files/2019-08/documents/2012-guidelines-water-reuse.pdf.

[34] García-Gallegos E., Hernández-Acosta E., García-Nieto E., Acevedo-Sandoval O.A. (2011). Lead content and translocation in oats (*Avena sativa*, L.) and broad bean (*Vicia faba*, L.) from contaminated soil. Rev. Chapingo Ser. Cienc. For. Ambiente.

[35] Ruiz-Huerta E.A., Armienta-Hernández M.A. (2012). Accumulation of arsenic and heavy metals in maize near mine tailings. Rev. Int. Contam. Ambient..

[36] Zhuang P., Zou B., Li N.Y. (2009). Heavy metal contamination in soils and food crops around Dabaoshan mine in Guangdong, China: Implication for human health. Environ. Geochem. Health.

[37] Liu H., Probst A., Liao B. (2005). Metal contamination of soils and crops affected by the Chenzhou lead/zinc mine spill (Hunan, China). Sci. Total Environ..

[38] Wang Q., Cai J., Gao F., Li Z., Zhang M. (2023). Pollution Level, Ecological Risk Assessment and Vertical Distribution Pattern Analysis of Heavy Metals in the Tailings Dam of an Abandon Lead–Zinc Mine. Sustainability.

[39] Ba V.N., Thien B.N., Phuong H.T., Hong-Loan T.T., Anh T.T. (2024). Bioconcentration and translocation of elements from soil to vegetables and associated health risk. J. Food Compost. Anal..

[40] Zhu Z., Yang X.D., Xu Z.Q., Fei J.C., Peng J.W., Rong X.M., Huang Y., Yang X.E. (2021). Foliar uptake, translocation and accumulation of heavy metals from atmospheric deposition in crops. J. Plant Nutr. Soil. Sci..

[41] National Water Commission National Meteorological System of Mexico. National Climatological Database..

[42] Yu P., Shao X., Wang M., Zhu Z., Tong Z., Peng J., Deng Y., Huang Y. (2024). Effects of atmospheric deposition on heavy metal contamination in paddy field systems under different functional areas in ChangZhuTan, Hunan Province, China. Sci. Total Environ..

[43] Ministry of the Environment and Natural Resources Mexican Official Standard NOM-141-SEMARNAT-2003, Which Establishes the Procedure for Characterizing Tailings, as Well as the Specifications and Criteria for the Characterization and Preparation of the Site, Project, Construction, Operation, and Post-Operation of Tailings Dams. https://www.profepa.gob.mx/innovaportal/file/1317/1/nom-141-semarnat-2003.pdf.

[44] Food and Agriculture Organization of the United Nations (FAO) and the World Health Organization (WHO) CAC/RCP 56-2004 Code of Practice for the Prevention and Reduction of Lead in Food. https://www.fao.org/input/download/standards/10099/CXP_056s.pdf.

[45] Lyu Z., Chai J., Xu Z., Qin Y., Cao J. (2019). A Comprehensive Review on Reasons for Tailings Dam Failures Based on Case History. Adv. Civil. Eng..

